# A mutant *wfs1* zebrafish model of Wolfram syndrome manifesting visual dysfunction and developmental delay

**DOI:** 10.1038/s41598-021-99781-0

**Published:** 2021-10-14

**Authors:** G. Cairns, F. Burté, R. Price, E. O’Connor, M. Toms, R. Mishra, M. Moosajee, A. Pyle, J. A. Sayer, P. Yu-Wai-Man

**Affiliations:** 1 International Centre for Life, Institute of Genetic Medicine, Newcastle University, Newcastle upon Tyne, UK; 2 Interdisciplinary School of Health Science, Faculty of Health Sciences, University of Ottawa, Ottawa, Canada; 3 Children’s Hospital of Eastern Ontario Research Institute, University of Ottawa, Ottawa, Canada; 4 UCL Institute of Ophthalmology, University College London, London, UK; 5 John van Geest Centre for Brain Repair and MRC Mitochondrial Biology Unit, Department of Clinical Neurosciences, University of Cambridge, Cambridge, UK; 6 Moorfields Eye Hospital NHS Foundation Trust, London, UK; 7 Great Ormond Street Hospital for Children NHS Foundation, Trust, London, UK; 8 The Wellcome Centre for Mitochondrial Research, Translational and Clinical Research Institute, Newcastle University, Newcastle upon Tyne, UK; 9 Department of Renal Medicine, Freeman Hospital, The Newcastle Upon Tyne Hospitals NHS Foundation Trust, Newcastle upon Tyne, UK; 10 National Institute for Health Research Newcastle Biomedical Research Centre, Newcastle upon Tyne, UK; 11 Cambridge Eye Unit, Addenbrooke’s Hospital, Cambridge University Hospitals, Cambridge, UK

**Keywords:** Genetics, Eye diseases

## Abstract

Wolfram syndrome (WS) is an ultra-rare progressive neurodegenerative disorder defined by early-onset diabetes mellitus and optic atrophy. The majority of patients harbour recessive mutations in the *WFS1* gene, which encodes for Wolframin, a transmembrane endoplasmic reticulum protein. There is limited availability of human ocular and brain tissues, and there are few animal models for WS that replicate the neuropathology and clinical phenotype seen in this disorder. We, therefore, characterised two *wfs1* zebrafish knockout models harbouring nonsense *wfs1a* and *wfs1b* mutations. Both homozygous mutant *wfs1a*^*−/−*^ and *wfs1*b^*−/−*^ embryos showed significant morphological abnormalities in early development. The *wfs1*b^*−/−*^ zebrafish exhibited a more pronounced neurodegenerative phenotype with delayed neuronal development, progressive loss of retinal ganglion cells and clear evidence of visual dysfunction on functional testing. At 12 months of age, *wfs1b*^*−/−*^ zebrafish had a significantly lower RGC density per 100 μm^2^ (mean ± standard deviation; 19 ± 1.7) compared with wild-type (WT) zebrafish (25 ± 2.3, p < 0.001). The optokinetic response for *wfs1b*^−/−^ zebrafish was significantly reduced at 8 and 16 rpm testing speeds at both 4 and 12 months of age compared with WT zebrafish. An upregulation of the unfolded protein response was observed in mutant zebrafish indicative of increased endoplasmic reticulum stress. Mutant *wfs1*b^*−/−*^ zebrafish exhibit some of the key features seen in patients with WS, providing a versatile and cost-effective in vivo model that can be used to further investigate the underlying pathophysiology of WS and potential therapeutic interventions.

## Introduction

Wolfram syndrome (WS) is a neurodegenerative disorder defined historically by a cluster of clinical manifestations, namely, diabetes insipidus, diabetes mellitus, optic atrophy and sensorineural deafness (DIDMOAD)^1–3^. It is now well established that a significant proportion of patients will develop additional neurological and psychiatric deficits such as ataxia, epilepsy and depression, renal tract abnormalities, and in some cases infertility^4–6^. The majority of patients with WS harbour recessive mutations within the *WFS1* gene (4p16, OMIM 606201), which encodes for the transmembrane endoplasmic reticulum (ER) protein Wolframin^1,7^. Wolframin is abundantly expressed in retinal, neuronal and muscle tissues^8^. It is a multifunctional protein that regulates a host of cellular functions including the dynamic interaction with mitochondria at mitochondria-associated membranes (MAMs)^9,10^. It negatively regulates the ER stress sensor ATF6α in β-islet cells through ubiquitination and proteasome-mediated degradation of the protein^11^. Wolframin also has roles in calcium ion homeostasis^9,12–15^, proinsulin modification^16^, the regulation of the cell cycle^17^, and calpain activation^18^.

The majority of experimental animal studies have been performed on mouse models of WS with a focus on the development of diabetes and ER stress, which is a key pathway implicated in the loss of β-islet cells^19^. A knockout mouse with disruption of exon 2 in the *Wfs1* gene developed progressive β-cell loss and glucose intolerance^20^. It has been suggested that an alternative glucose clearing pathway through the urine exist in mice protecting them from a full diabetic phenotype^20,21^. Testing of visual function of the exon 2, *Wfs1* knockout mouse showed only mild changes with no differences in visual function compared with the early-onset optic atrophy phenotype seen in patients with WS^22^. A rat model of WS has recently been characterised (*Wfs1*-ex5-KO232) with features of diabetes mellitus and a reduction in brain medullary volume, mirroring the neurodegeneration observed in patients with WS^23^. This rat model also developed retinal gliosis and cataracts with optic nerve volume reductions and disturbed myelin structure^23^. A more basic *Drosophila* model with knockdown of *wfs1* has been generated that showed behavioural deficits, neurodegeneration and a reduced lifespan^24^.

Visual loss in WS is progressive, starting in early childhood, and it is an important cause of registrable blindness in children and young adults^25^. Research into WS has been limited by the lack of access to diseased human tissues, in particular retinal, optic nerve and brain samples. Zebrafish models are frequently used to study inherited ocular and central nervous system disorders as the embryos are amenable to germline genetic manipulation or more transient regulation of gene expression with morpholinos. Another distinct advantage is that the embryos are optically transparent, allowing easy visualisation of neuronal development in vivo, and there is great similarity in anatomical structures with humans. Using a number of whole-system and live imaging techniques, it is also possible to monitor and quantify changes during early development that would otherwise be more technically challenging and costly in murine models. Morpholinos have been used to transiently knockdown genes in zebrafish thought to be involved in the regulation of β-cell mass and the development of type 2 diabetes mellitus, including *wfs1*^26^. To our knowledge, no zebrafish with germline genetic knockout of *wfs1* has been reported that replicates the clinical phenotype seen in patients with WS.

Here, we describe a novel zebrafish model of WS and examine the role played by Wolframin in early development and neurodegeneration. Due to a genome duplication, *WFS1* has two orthologues in zebrafish, namely, *wfs1a* and *wfs1b*. Our findings indicate that *wfs1b*^*−/−*^ mutant zebrafish show disturbed neuronal development and progressive loss of retinal ganglion cells (RGCs) with impaired visual function.

## Results

### wfs1a^−/−^ and wfs1b^−/−^ zebrafish models

The sequences of the zebrafish orthologues *wfs1a* and *wsf1b* were compared with the human *WFS1* gene. The *wfs1a* and *wfs1b* sequences had 53.19% and 53.97% sequence homology with the human *WFS1* gene, respectively (Fig. S1A,B). The expression of *wfs1* in wild-type (WT) zebrafish was examined using reverse transcriptase PCR (RT-PCR). *wfs1b* is expressed at the one-cell stage suggesting possible maternal expression and it is constitutively expressed. *wfs1a* expression begins at a later time point only at 24 h post-fertilisation (hpf) (Fig. S1C). Quantitative RT-PCR (qRT-PCR) was used to determine the expression levels of the zebrafish orthologues *wfs1a* and *wfs1b* in tissue lysates from 4-month-old zebrafish. *wfs1a* is more highly expressed in muscle compared with *wfs1b*. *wfs1b* is highly expressed in the eye and the brain, whereas *wfs1a* shows low levels of expression in these two tissues (Fig. S1D,E).

Zebrafish with germline mutations in *wfs1a* and *wfs1b* were crossed to create single homozygous *wfs1a*^*−/−*^ and *wfs1b*^*−/−*^ lines, and a double knockout *wfs1a*^*−/−*^*b*^*−/−*^ zebrafish (Fig. S2).

### Morphological assessment of wfs1a^−/−^ and wfs1b^−/−^ zebrafish

**Figure 1:**
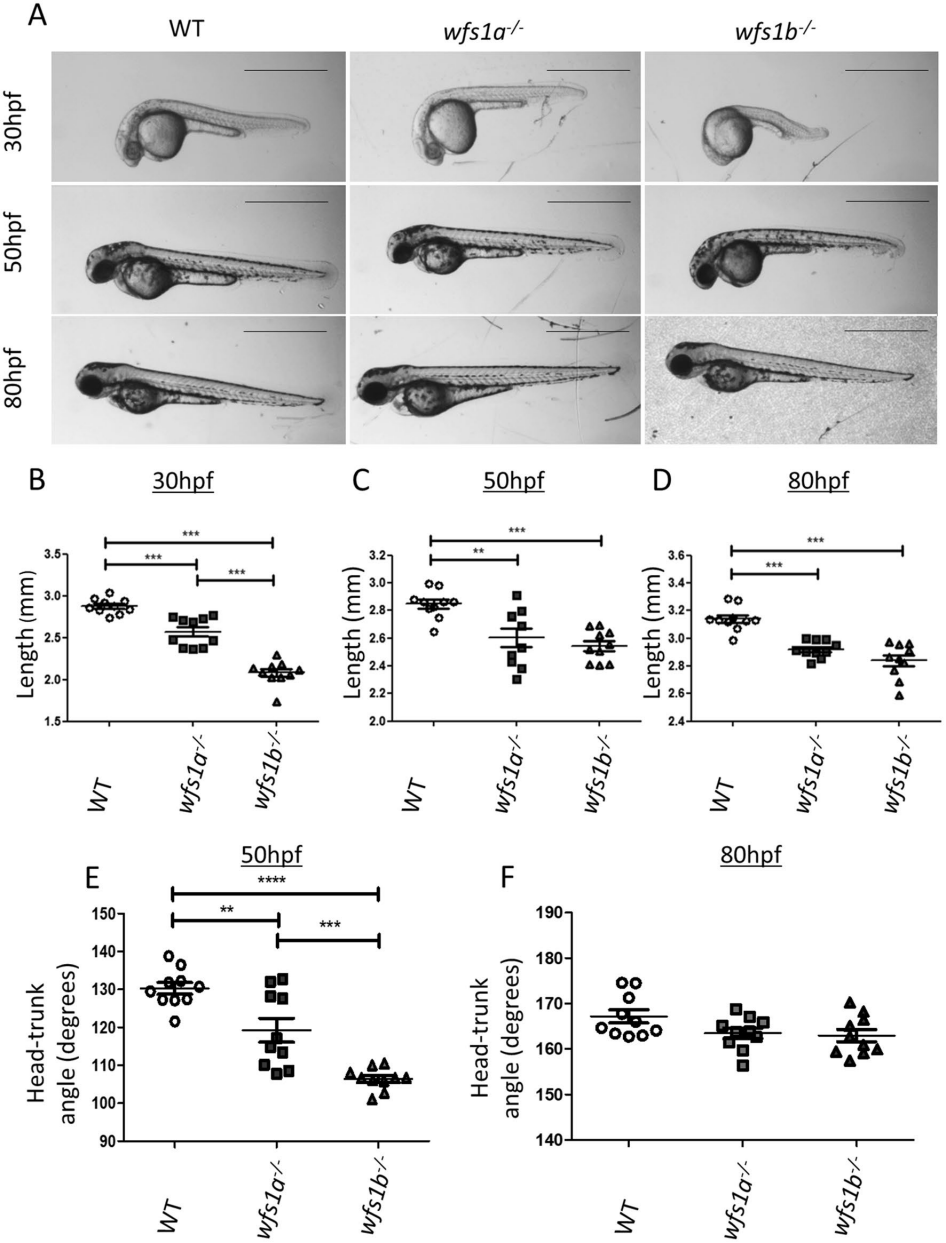
Phenotypic analysis of *wfs1a*^*−/−*^* and wfs1b*^*−/−*^ zebrafish. (**A**) Zebrafish were imaged at 30, 50 and 80 hpf. Scale bars represent 1 mm. (**B–D**) Zebrafish length at 30 hpf (**B**), 50 hpf (**C**) and 80 hpf (**D**). (**E,F**) Zebrafish head-trunk angles at 50 hpf (**E**) and 80 hpf (**F**). Zebrafish length and head tail angle was measured using ImageJ and data plots represent mean ± SEM (n = 10). Statistical significance was determined by One-Way ANOVA with Bonferroni multiple comparisons. **p < 0.01; ***p < 0.001; *hpf* hours post-fertilisation, *SEM* standard error of the mean.

The early embryonic development of *wfs1a*^−/−^ and *wfs1b*^−/−^ zebrafish was assessed morphologically at 30, 50 and 80 hpf (Fig. 1A). Developing *wfs1a*^*−/−*^ and *wfs1b*^*−/−*^ embryos were significantly shorter compared with WT embryos at all time points (Fig. 1B–D). This developmental delay was accompanied by a significant change in head-trunk angle at 50 hpf, which was restored by 80 hpf (Fig. 1E,F). There were no significant differences in eye area between wild-type (WT), *wfs1a*^*−/−*^ and *wfs1b*^*−/−*^ embryos at 80 hpf when normalised to length (Fig. S5).

### Heat shock response of wfs1a^−/−^ and wfs1b^−/−^ zebrafish

**Figure 2:**
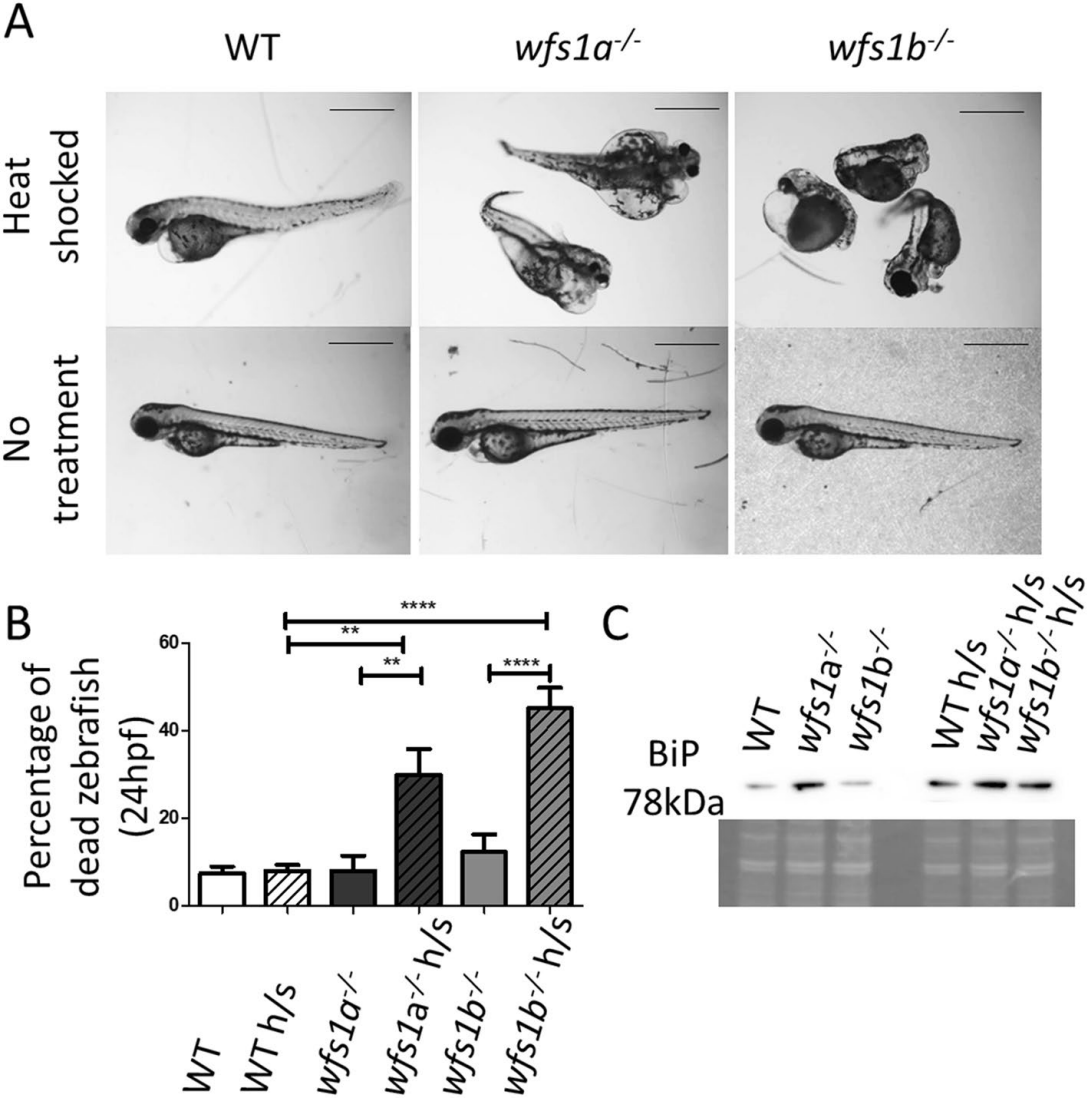
Effects of the unfolded protein response in *wfs1a*^*−/−*^ and *wfs1b*^*−/−*^ zebrafish. Treated zebrafish were heat shocked for 1 h at 6 hpf (groups of 50 embryos). (**A**) Representative images of the morphological effects for each genotype at 80 hpf. Scale bar = 1 mm. (**B**) Percentage of dead zebrafish at 24 hpf. Data plots represent mean ± SEM (n = 5 groups of 50). Statistical significance was determined by One-Way ANOVA with Bonferroni multiple comparisons. (**C**) Immunoblot of BiP in untreated and heat shocked zebrafish showing an upregulation of BiP in response to heat shock. Coomassie staining demonstrates equal loading. *p < 0.05; **p < 0.01; ****p < 0.0001; h/s: heat shock treated.

*wfs1a*^*−/−*^* and wfs1b*^*−/−*^ zebrafish were heat shocked to increase the amount of unfolded proteins in the ER and to examine for aberrations in the unfolded protein response (UPR), which is a hallmark of ER stress. Heat shocking resulted in severe morphological changes in the tail curvature and eye development, and in the formation of prominent cardiac oedema (Fig. 2A). Significantly higher levels of death were observed in the *wfs1a*^*−/−*^ and *wfs1b*^*−/−*^ zebrafish compared to WT (Fig. 2B). Heat shock treatment of *wfs1a*^*−/−*^ and *wfs1b*^*−/−*^ zebrafish stimulated the UPR, as evidenced by increased BiP, which is the master regulatory of the UPR (Fig. 2C). Although BiP expression seemed increased in *wfs1a*^*−/−*^ zebrafish without heat shock treatment, this was not a consistent finding in repeat experiments. Quantitative PCR analysis of BiP expression in tissue lysates at 48 hpf showed no significant differences between WT, *wfs1*a^*−/−*^ and *wfs1*b^*−/−*^ zebrafish (Fig. S6).

### Neuronal development of wfs1a^−/−^ and wfs1b^−/−^ zebrafish

**Figure 3:**
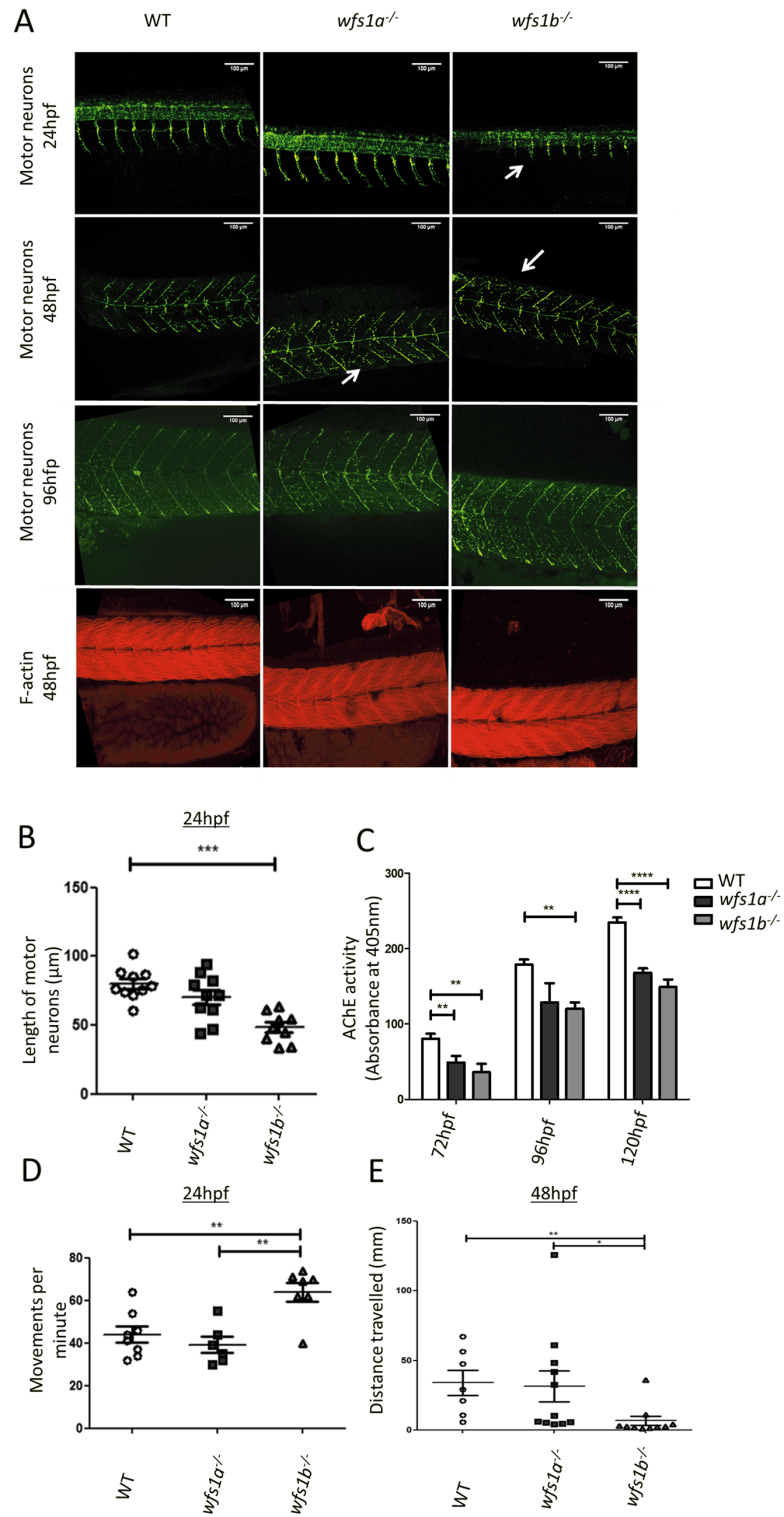
Neuronal development in *wfs1a*^*−/−*^ and *wfs1b*^*−/−*^ zebrafish. (**A**) Immunofluorescence of motor neurons (SV-2 stained using anti-SV2 antibody in green) and muscle fibres (F-actin stained using phalloidin in red). Shorter or missing neurons are highlighted with white arrows. (**B**) Quantification of the length of motor neurons in 24 hpf zebrafish (WT n = 10; *wfs1a* n = 11; *wfs1b* n = 9). For each fish, 9–10 neurons were measured and the average length was calculated. (**C**) Acetylcholine esterase (AChE) activity assay of developing zebrafish larvae (3–5 dpf). (**D**) Coiling response of zebrafish embryos at 24 hpf. The average movement per fish per minute was calculated from ~ 15 embryos (WT n = 8; *wfs1a* n = 6; *wfs1b* n = 7) (Supplementary Video 1). (**E**) Quantification of the touch response of zebrafish embryos at 48 hpf. The distance travelled was recorded in response to tactile stimulation (WT n = 8; *wfs1a* n = 11; *wfs1b* n = 10) (Supplementary Video 2). Data plots represent mean ± SEM. Statistical significance was calculated using One-way ANOVA with Bonferroni’s multiple comparison tests. **p < 0.01; ***p < 0.001; ****p < 0.0001; *dpf* days post-fertilisation.

Whole-mount immunofluorescence studies of zebrafish tail motor neurons were performed to visualise neuronal development in wfs1a^−/−^ and wfs1b^−/−^ zebrafish. Shorter or absent motor neurons were observed in the tail region in both *wfs1a*^*−/−*^ and *wfs1b*^*−/−*^ zebrafish (Fig. 3A). The length of the motor neurons within the tail of *wfs1b*^*−/−*^ zebrafish was significantly shorter compared with WT zebrafish at 24 hpf (p < 0.005, Fig. 3B). At 48 hpf, there was no significant difference in the length of motor neurons between *wfs1a*^*−/−*^, *wfs1b*^*−/−*^ and WT zebrafish.

Phalloidin staining of filamentous (F)-actin showed normal structural integrity of the tail musculature. At 4 days post-fertilisation (dpf), the neuronal axonal segments that were absent along the myosepta during early development were seen to extend in the appropriate location (Fig. 3A). Reduced acetylcholinesterase (AChE) activity was observed at 3, 4 and 5 dpf (Fig. 3C).

To determine whether the observed disruption in neuronal development was reflected at a functional level, the motor behaviours of the zebrafish were tested using coiling and touch response assays. At 24 hpf, zebrafish perform spontaneous coiling movements (coiling response). A significant increase in the occurrence of these coiling movements was observed in *wfs1b*^*−/−*^ zebrafish compared with *wfs1a*^*−/−*^ and WT zebrafish (Fig. 3D, Supplementary Video 1). By 48 hpf, zebrafish have developed the ability to respond to tactile stimulation by swimming rapidly away from the applied stimulus (touch response assay). The touch response in terms of distance travelled was significantly decreased in *wfs1b*^*−/−*^ zebrafish compared with *wfs1a*^*−/−*^ and WT zebrafish (Fig. 3E, Supplementary Video 2).

### Retinal structure and visual function in wfs1a^−/−^ and wfs1b^−/−^ zebrafish

**Figure 4:**
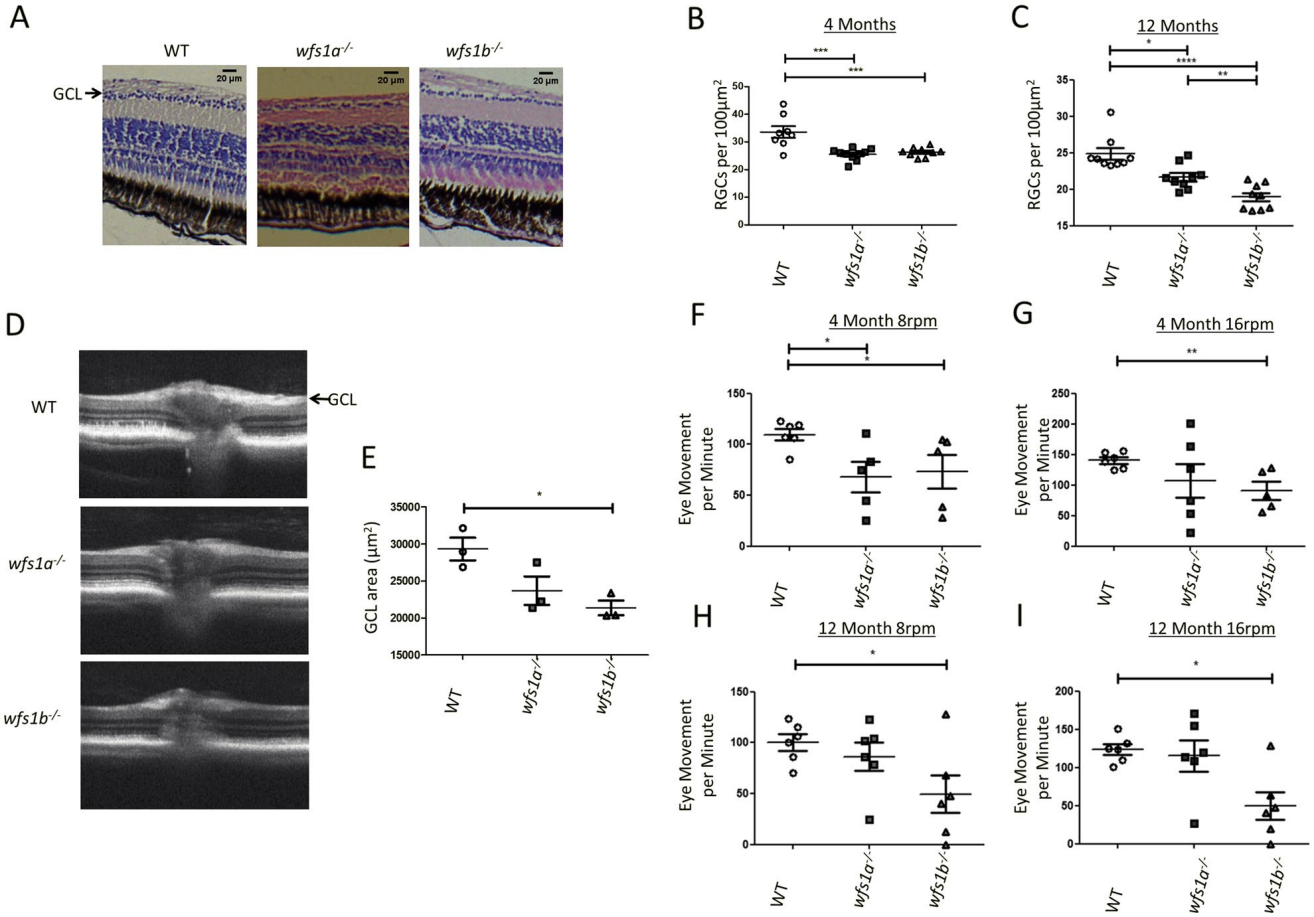
Retinal ganglion cell count and visual function in *wfs1a*^*−/−*^ and *wfs1b*^*−/−*^ zebrafish. (**A**) Representative images of WT, *wfs1a*^*−/−*^* and wfs1b*^*−/−*^ retinal sections at 4 months of age (scale bar = 20 µm). For RGC counts, 6 boxes of the same area (100 μm^2^) were used with 3 boxes on either side of the optic nerve. The number of RGC cell bodies were counted and averaged. (**B**) RGC count per 100 μm^2^ at 4 months of age (WT n = 8; *wfs1a*^*−/−*^ n = 10; *wfs1b*^*−/−*^ n = 9). (**C**) RGC count per 100 μm^2^ at 12 months of age (WT n = 10; *wfs1a*^*−/−*^ n = 9; *wfs1b*^*−/−*^ n = 9). Data plots represent mean ± SEM. Statistical significance was calculated using the Kruskal–Wallis test (One-way ANOVA on ranks). (**D**) Optical coherence tomography (OCT) images of retinal cross-sections from 12-month-old zebrafish showing significant thinning of the ganglion cell layer (GCL) in *wfs1a*^*−/−*^* and wfs1b*^*−/−*^ zebrafish (arrow). (**E**) Measurement of GCL area (WT mean = 29,393 ± 2653 μm^2^; *wfs1a*^*−/−*^ mean = 23,688 ± 3332 μm^2^; *wfs1b*^*−/−*^ mean = 21,363 ± 1,737 μm^2^; n = 3 for all 3 groups). Data plots represent mean ± SEM. Statistical significance was calculated using One-way ANOVA with Bonferroni’s multiple comparison tests. (**F–I**) Optokinetic response (OKR) of 4- and 12-month-old fish tested at 8 rpm and 16 rpm. Videos were recorded of fish eye tracking and the movements were manually counted (Supplementary video 3). Data plots represent mean ± SEM. Statistical significance was calculated using One-way ANOVA with Bonferroni’s multiple comparison tests. *p < 0.05; **p < 0.01; ***p < 0.005; ****p < 0.001; rpm: revolutions per minute.

Histological analysis of *wfs1a*^−/−^ and *wfs1b*^−/−^ zebrafish retinas revealed a significant loss of RGCs at 4 months of age (Fig. 4A,B). The RGC density per 100μm^2^ (mean ± standard deviation) was 34 ± 5.9 for WT zebrafish, 26 ± 2.1 for *wfs1a*^*−/−*^ zebrafish, 27 ± 1.7 for *wfs1b*^*−/−*^ zebrafish. At 12 months of age, *wfs1b*^*−/−*^ zebrafish had a significantly lower RGC density (19 ± 1.7) compared with WT zebrafish (25 ± 2.3) (Fig. 4C). Optical coherence tomography (OCT) imaging showed thinner RGC layers in *wfs1a*^*−/−*^ and *wfs1b*^*−/−*^ zebrafish compared with WT zebrafish at 12 months of age (Fig. 4D). The GCL area was significantly thinner in *wfs1b*^*−/−*^ zebrafish compared with WT zebrafish (Fig. 4E). A non-significant trend was observed for *wfs1a*^*−/−*^ zebrafish compared with WT zebrafish.

In order to investigate the effects on visual function, optokinetic response (OKR) tests were performed at 4 and 12 months of age (Fig. 4F–I, Supplementary Video 3). Zebrafish eye movements were analysed in response to a rotating black and white grating at different rotations per minute (rpm). The OKR for *wfs1b*^−/−^ zebrafish was significantly reduced at 8 and 16 rpm testing speeds at both 4 and 12 months of age. In comparison, the OKR for *wfs1a*^*−/−*^ zebrafish was not significantly different compared with WT zebrafish at 4 months of age (16 rpm) and 12 months of age (8 and 16 rpm).

### Fertility of wfs1a^−/−^ and wfs1b^−/−^ zebrafish

**Figure 5:**
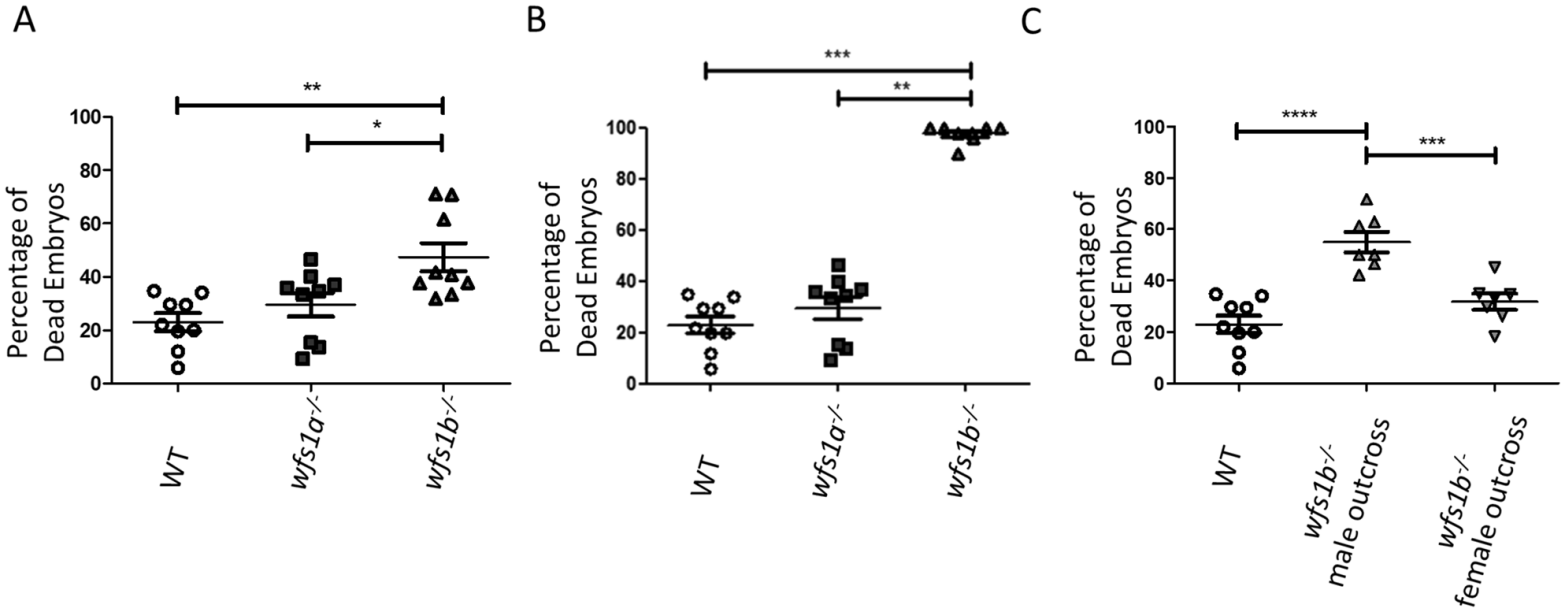
Fertility of *wfs1a*^*−/−*^ and *wfs1b*^*−/−*^ zebrafish. (**A**) Percentage of dead embryos at 24 hpf produced from adults < 9 months (n = 9). (**B**) Percentage of dead embryos at 24 hpf produced from adults > 9 months (n = 9). (**C**) Percentage of dead embryos at 24 hpf in randomly selected embryos from *wfs1b*^−/−^ zebrafish (male or female) that were outcrossed to WT (controls n = 9, *wfs1b*^−/−^ outcrosses n = 7). A total of 50 randomly selected embryos were placed in E3 medium, incubated overnight and any dead embryos were determined the next morning. Data plots represent mean ± SEM. Statistical significance was determined by One-Way ANOVA with Bonferroni multiple comparisons. **p < 0.01; ***p < 0.001; ****p < 0.0001.

A significantly higher number of unfertilised embryos was observed in *wfs1b*^*−/−*^ zebrafish that were < 9 months old compared with *wfs1a*^*−/−*^ and WT zebrafish (Fig. 5A). In more aged zebrafish (> 9 months old), a further drop in fertility was observed for *wfs1b*^*−/−*^ zebrafish, with a mean of 97.8% of embryos remaining unfertilised (Fig. 5B). When *wfs1b*^*−/−*^ zebrafish were outcrossed with WT zebrafish, a high percentage of dead embryos were seen in the *wfs1b*^*−/−*^ male outcross, but not in the *wfs1b*^*−/−*^ female outcross (Fig. 5C). Double mutants (*wfs1*a^−/−^*b*^−/−^) were unable to breed indicating significant infertility.

The significantly higher number of unfertilised embryos in *wfs1b*^*−/−*^ knockouts could be due to fertilised embryos dying within the first 24 h. To determine if that was the case, eggs were collected at 8 hpf and unfertilised embryos were removed. The percentage of dead embryos was then assessed at 24 hpf. No significant difference was observed between fertilised controls (mean = 5.6%), *wfs1*a^*−/−*^ knockouts (mean = 6.4%), and *wfs1*b^*−/−*^ knockouts (mean = 11.1%) (Fig. S7).

## Discussion

Zebrafish has been successfully applied to characterize disease mechanisms for rare inherited diseases and they have also proven useful as in vivo models for therapeutic screening. This is of particular relevance to WS caused by recessive *WFS1* mutations, which is a syndrome characterized by progressive visual loss from early childhood secondary to the irreversible loss of RGCs and optic nerve degeneration. As the zebrafish eye is comparable to the human eye^27,28^, and optic atrophy is a defining clinical feature of WS^29^, we investigated the morphological and functional characteristics in embryos and adult fish established from two mutant lines carrying stop codon mutations in *wfs1a* and *wfs1*b, which are the orthologues of *WFS1* in zebrafish.

Homozygous mutant *wfs1a*^*−/−*^ and *wfs1*b^*−/−*^ embryos showed significant developmental delay as judged by their morphology. This has been corroborated in culture models where impaired cell cycle progression has been observed in β-islet cells of a *wfs1* knockout mouse^16^. Cell cycle delay, especially in the early stages, could slow the development of the zebrafish embryo by reducing the rate of cell division. In addition to changes in the cell cycle, a greater susceptibility to apoptosis was observed in β-islet cells from this mutant mouse model, which was linked to an increased ER stress response^17^. We observed increased death rates in both *wfs1a*^*−/−*^ and *wfs1b*^*−/−*^ zebrafish and upregulation of the UPR as a response to heat shock treatment, pointing towards an increased ER stress response^30^. Changes in the UPR pathway have been well characterized in rat, mouse and *Drosophila* knockout models, as well as in human cell models of WS^17,23,24,31^. It will be important to explore as part of future mechanistic studies whether *wfs1*b^*−/−*^ zebrafish exhibit increased apoptosis and its contribution to the development of the neurodegenerative phenotype, in particular the loss of RGCs and visual dysfunction.

Over 50% of patients with WS will develop significant neurological complications, in particular cerebellar ataxia, peripheral neuropathy and spasticity due to pyramidal tract dysfunction^5^. As part of our phenotyping protocol, we investigated whether neuromuscular development was impaired in *wfs1a*^*−/−*^ and *wfs1b*^*−/−*^ zebrafish. Both models showed impairment in early development of motor neurons, but this defect was more pronounced in the *wfs1b*^*−/−*^ zebrafish. The dorsal growth along the vertical myoseptum was delayed with motor neurons not extending along a number of myosepta until later in development (4 dpf). Similar results were obtained in *Wfs1* shRNA-transfected mouse cortical neurons, which showed an improvement in mature neuron growth after an initial delay during early development^9^. It should be stressed that the developmentally delayed mutant embryos were not developed to the same stage prior to analysing motor neurons and measuring axon length in the tail region. Additional experiments comparing mutant and WT zebrafish that have reached similar developmental time points will provide further insight into the contribution of developmental delay to the observed neuronal defects.

The descending motor axons are affected in a subgroup of patients with WS resulting in spasticity^4–6^. In this study, we measured axon length in the tail region to assess general neuronal development in mutant zebrafish and to first establish that the defects in locomotion are indeed neural in origin and not due to the other factors, such as improper muscle development. The neural circuit for locomotion in the zebrafish is well characterised. It is controlled by the reticulospinal network in the hind brain, which is primarily composed of a pair of neurons, namely, the Mauthner (M) cells and the spiral fibre neurons. The M cells and its associated motor neurons running towards the tail form an intricate network that is activated in response to different types of stimuli (touch, sound and visual), ultimately controlling the movement of the zebrafish. Thus, it was important to look at the motor neurons in the tail region first to detail any possible defects and to confirm that the defects are neural in origin. Further work is needed to conduct more extensive analysis of the reticulospinal network in *wfs1b*^−/−^ zebrafish.

AChE is expressed in the central nervous system, peripheral cholinergic neurons and muscle fibres of the zebrafish embryo^32,33^. By measuring its activity in homogenates of zebrafish embryos, this assay provides an indirect measure of AChE, which correlates with the amount of neuronal tissue and the overall developmental stage in zebrafish^34^. A decrease in AChE activity was found in both *wfs1a*^*−/−*^ and *wfs1b*^*−/−*^ zebrafish, with the latter manifesting a more severe reduction. Phalloidin staining of filamentous (F)-actin showed normal structural integrity of the muscle fibres in the tail musculature, indicating that neuronal outgrowth is impaired without an underlying primary muscle fibre disorder. To determine whether the observed disruption in neuronal development was functionally relevant, the motor behaviours of the zebrafish were quantified using the coiling and touch response assays^35,36^. Both responses were significantly disrupted in *wfs1b*^*−/−*^ zebrafish, consistent with a developmental delay, and indicating that a lack of Wolframin likely affects neuronal development, contributing to the neurological deficits seen in patients with WS. Defective mitophagy has been implicated as the mechanism by which neuronal development is delayed in neurons from Wfs1-deficient mice, with shRNA silencing of the mitophagy-related proteins PINK1 and Parkin correcting this defect^9^. Further investigation of mitochondrial function and mitophagy in our zebrafish model is needed to provide a better understanding of the mechanisms involved in delayed neuronal development.

A pathological hallmark of WS is progressive RGC loss resulting in optic atrophy and visual failure in affected patients^37^. A significant reduction in RGC density was observed in both *wfs1a*^*−/−*^ and *wfs1b*^*−/−*^ models, with the loss of RGCs being more prominent in *wfs1b*^*−/−*^ zebrafish at 12 months implying a more severe degenerative process compared with the *wfs1a*^*−/−*^ zebrafish. OCT imaging confirmed marked thinning of the RGC layer and this was correlated with the reduced visual function recorded using the OKR in *wfs1b*^*−/−*^ zebrafish. Our data show that zebrafish lacking the *wfs1b* orthologue is an attractive model that successfully recapitulates the progressive RGC loss and visual dysfunction seen in patients with WS. Research into this relatively rare inherited form of optic atrophy has been limited by the lack of human tissues and the *wfs1b*^*−/−*^ zebrafish will be a useful resource to dissect the disease mechanisms that precipitate RGC loss in this disorder.

WS is a multisystemic neurodegenerative disorder and reduced fertility has also been reported in some patients^38,39^. Although this observation needs to be investigated further, our data indicate that the fertility of adult fish lacking *wfs1b* is impaired with an increased number of unfertilised embryos. When adults older than 9 months of age were outcrossed with WT zebrafish, they were able to produce viable offspring. However, the *wfs1b*^*−/−*^ males still exhibited a significantly higher percentage of unfertilised offspring. Consistent with our findings, male knockout mice with deleted *Wfs1* gene have reduced fertility due to abnormal sperm morphology and reduced number of spermatogenic cells^40^.

Wolframin plays an important role in early zebrafish development and *wfs1b*^*−/−*^ zebrafish exhibit a more severe neurological and ocular phenotype compared with the *wfs1a*^*−/−*^ zebrafish. This could be due to their tissue-specific expression patterns with *wfs1b* being constitutively expressed with high levels in the eye and brain compared with *wfs1a,* which is predominantly expressed in muscle. Interestingly, skeletal muscle seems to be spared in WS with myopathy not being reported in patients with confirmed pathogenic *WFS1* mutations^5^.

In summary, we have characterized two *wfs1* zebrafish knockout models with the *wfs1b*^*−/−*^ zebrafish recapitulating some of the key ocular and neurological deficits observed in patients with WS. This zebrafish model will be a valuable tool to further investigate the pathophysiology in WS and the pathways that could potentially be modulated to delay or stop the neurodegenerative process driving cellular loss in this disorder. WS is an important cause of blindness in children and young adults. There are currently no effective treatments available and the progressive loss of RGCs and visual dysfunction observed in the *wfs1b*^*−/−*^ zebrafish provide powerful readouts for drug screening and investigating new therapeutic interventions.

## Methods

All the methods have been reported in accordance with the ARRIVE guidelines (https://arriveguidelines.org).

### Zebrafish maintenance

All zebrafish procedures were performed under Home Office UK licence regulations and approved by the Newcastle University Animal Welfare and Ethical Review Board. Fish strains used in this study include AB, sa10021 (*wfs1a*) and sa16422 (*wfs1b*). Embryos were collected from breeding pairs of zebrafish and grown for up to 5 days in E3 medium (5 mM NaCl, 0.17 mM KCl, 10 mM HEPES, 0.33 mM MgSO_4_, 0.33 mM CaCl_2_ 0.00002% methylene blue) placed in an incubator at 27.5 °C.

### Zebrafish strains

Two heterozygote mutant lines were purchased from the European Zebrafish Resource Centre (EZRC). The *wfs1a (wfs1a*^*sa10021*^^41^) line had a G > A nonsense mutation at amino acid (aa) 692 resulting in a TAG stop codon (Fig. S3). The *wfs1*b (*wfs1b*^*sa16422*^^41^) line had a G > A nonsense mutation at aa 493 resulting in a TGA stop codon. The F2 lines obtained from the EZRC were outcrossed twice prior to experimental work. The lines were inbred to create two homozygous lines, *wfs1a*^*−/−*^ and *wfs1*b^*−/−*^ (Fig. S2). This was confirmed by Sanger sequencing of isolated genomic DNA (Fig. S2A,B). Unless stated, experimental crosses were performed with homozygous knockouts to remove the confounding factor of maternal RNA. Zebrafish were group mated with 3 males and 3 females per large breeding tank. Double knockout mutants were also derived (Fig. S2C). Experimental blinding was not performed. However, prior to analysis, data was blinded to minimise potential bias. AB control lines were used in all experiments (https://zfin.org/action/genotype/view/ZDB-GENO-960809-7).

### Zebrafish imaging

Imaging was carried out using bright field microscopy. Images were taken on a Leica MZ16F stereomicroscope with a Leica DFC420 C camera attachment on the Leica Application Suite V3 program. Zebrafish measurements were performed using ImageJ. A micrometer image at each magnification was used to set the scale. Zebrafish length measurements at 30 hpf used straight lines from head to tail. At 50 hpf and 80 hpf, measurements were taken from the first muscle somite to the tip of the tail. Head-trunk angles were assessed and quantified using the angle tool from ImageJ as described previously^42^. The segmented line tool in ImageJ was used to measure axon length (Fig. S4).

### Sequencing

Genomic DNA was isolated using the Hotshot method^43^. DNA was amplified by PCR using the following primers:*wsf1a*_F ACCCCAATCAGACACACCTT, *wsf1a*_R ATCGAGTCCAGAGTCGCAGT, *wfs1b_*F AGCCATACCTCTACTTTCTCCT and *wfs1b_*R AGATGCACACTGTTACGATCA using Mytaq (Bioline). PCR reactions were purified by ExoFastAP reaction to remove any excess nucleotides that could interfere with Sanger sequencing. The purified products were then subjected to a BigDye terminator cycle sequencing reaction v3.1 (Applied Biosystems). The big dye reaction was purified by ethanol precipitation, resuspended in HiDi (Applied Biosystems) and then sequenced using a capillary electrophoresis on a 3130xl Genetic Analyser (Applied Biosystems).

### Immunofluorescence of whole-mount zebrafish

Zebrafish were manually dechorionated and euthanized in 4 mg/ml buffered tricaine methanesulfonate (MS222), diluted 1:1 in system water. Whole-mount staining was performed as previously described^44^. Mouse anti-SV2 antibody was applied at a 1:200 dilution (Developmental Studies Hybridoma Bank) and Alexa Fluor™ 488 (ThermoFisher) anti-mouse secondary antibody was used at a 1:1000 dilution. F-actin staining was performed using Alexa Fluor™ 594 Phalloidin (ThermoFisher) at a 1:1000 dilution. Zebrafish were imaged on a Nikon A1R confocal microscope at a 20× objective (NA 0.75) and *z*-stack images were obtained.

### Larvae tracking

At 24 hpf and 27 hpf, zebrafish were imaged using a Leica stereomicroscope with a Chameleon digital camera (CMLN-13s2M) 25 frames per second. Starting at 17 hpf, zebrafish start to perform spontaneous coiling movements (coiling response), which refers to a movement of the tail in the chorion, and this gradually decreases in frequency until it stops altogether at 27 hpf (35, 36). Spontaneous coiling movements were counted per embryo over a period of one minute. Zebrafish tracking was performed as described previously^45^. Briefly, the 48 hpf touch response was recorded using a Canon legria hfr76 camera at 25 frames per second. Single embryos were placed in E3 medium and then touched on the back of the head with a fine pipette tip. Videos were analysed using Trackmate (ImageJ).

### Immunoblot

Zebrafish embryos/tissues were lysed in RIPA buffer with protease inhibitor tablet (Roche) using a Tissue Ruptor. Lysates were maintained at 4 °C for 30 min before centrifugation at 13,000×*g* for 15 min. The supernatant was quantified with the Bradford assay. 50 μg of protein was loaded according to the NuPAGE Bis–Tris Mini Gels protocol (ThermoFisher) and transferred using iBlot™ 2 Transfer Stacks, PVDF, mini (ThermoFisher) according to manufacturer’s instructions. PVDF membranes were blocked in 5% low-fat dried milk in Tris-buffered saline (TBS) and 0.1% Tween 20 (TBS-T) for 1 h at room temperature, and then incubated with primary antibody (in 5% milk TBS-T), Anti-HSPA5 1:1000 dilution (Abnova PAB2462), overnight at 4 °C. Blots were washed with TBS-T and anti-rabbit polyclonal HRP conjugated antibody (Agilent, Santa Clara, USA) secondary antibody in 5% milk TBS-T was added for 1 h. SeeBlue Plus2 Pre-stained protein standard was used (ThermoFisher). The blots were visualised using Biorad Clarity ECL and imaged with an Amersham Imager 600.

### Histology

Adult zebrafish were euthanized in 4 mg/ml buffered tricaine methanesulfonate (MS222), diluted 1:1 in system water, before being decapitated. Their heads were then fixed for 10 days at 4 °C in 4% paraformaldehyde. Decalcification was performed as described previously^46^. The tissue was dehydrated in increasing grades of ethanol (70%, 90% and 100%), cleared in xylene and impregnated with paraffin wax. Zebrafish heads were embedded in paraffin, sectioned at 4 μm using a Leica RM 2135 microtome (Leica Biosystems) and then subjected to haematoxylin and eosin staining. Images were acquired by light microscopy using an Axio Imager Z1 fluorescence microscope (Zeiss).

### Heat shock treatment

The UPR, which is a hallmark of ER stress, is regulated by Wolframin^11^. Heat shocking increases the amount of unfolded proteins in the ER allowing for the examination of the UPR. For inducing the UPR, zebrafish were heated to 37.5 °C in E3 medium for 1 h. Zebrafish were euthanised in 4 mg/ml buffered tricaine methanesulfonate (MS222), diluted 1:1 in system water. To anesthetise zebrafish, a 1:20 dilution was used in system water. Zebrafish were group mated with 3 males and 3 females per large breeding tank. Zebrafish were exposed to 1 h of heat shock treatment at 37 °C to induce protein misfolding^30^.

### Optokinetic response (OKR)

Zebrafish were anaesthetized, immobilised in a foam holder within a petri dish filled with tank water and then placed into a custom-made optokinetic device^47^. This device included a 12 cm rotating optokinetic drum with adjustable speeds and stereo microscope (Zeiss Stemi-2000C) c-mounted with a digital SLR camera at 30 fps (Nikon D5100). The distance between the zebrafish eye and the rotating drum was 6.5 cm. When the fish had regained consciousness, the drum was rotated for 30 s clockwise and 30 s anti-clockwise, using a grating width of 0.8 cm. The rotational speeds used were 8 rpm and 16 rpm, corresponding to angular speeds of 48 degrees per second and 96 degrees per second, respectively. The eye movements were counted manually from video recordings.

### Optical coherence tomography (OCT)

OCT images were captured using the Bioptigen Envisu R2200 SDOIS (Bioptigen, Inc., Morrisville, USA)^48^. The zebrafish were anesthetised in a 1:20 tricaine to system water ratio and placed in a rubber holder. For optic nerve imaging, a 1.4 × 1.4 mm perimeter protocol with 1000 A-scans per B-scan with 100 total scans was used. Images were created using ImageJ. The ganglion cell layer (GCL) was defined manually using ImageJ. The combined GCL area was then determined for the region extending from the optic disc to a radius of 400 µm.

### RNA extraction and cDNA synthesis

RNA was extracted using a combined method of TRIzol (Invitrogen) and RNeasy kit (Qiagen). About 50 mg of tissue was homogenised in TRIzol and incubated at room temperature for 5 min. 1:5 ratio of chloroform to TRIzol was added and mixed before centrifugation at 12,000×*g* for 15 min. The aqueous phase was removed and added to 70% ethanol at a 1:1 ratio. This was then added to an RNeasy spin column as per the manufacturer’s instructions for the remainder of the protocol. cDNA synthesis was performed using the Applied Biosystems: High-Capacity cDNA Reverse Transcription Kit with 1000 ng RNA.

### Quantitative PCR (qPCR)

qPCR was performed using qPCR iQ™ SYBR^®^ Green Supermix (BioRad) according to the manufacturer’s instructions. The following primers were used with an annealing temperature of 58 °C: (i) *wfs1a*: wfs1a-qF 5′-TGTGCCCTGTGTGCTCTAC-3′ and wfs1a-qR 5′-GGCAACACAAGTACGGATCA-3′; (ii) *wfs1b:* wfs1b-qF 5′-CGCCCCGAATCTAAGCTTTT-3′ and wfs1b-qR 5′-GCGGAAGTGTGTGTTTGTCT-3′; and (iii) *ef1α*: ef1αF 5′-CTGGAGGCCAGCTCAAACAT-3′ and ef1αR 5′-ATCAAGAAGAGTAGTACCGCTAGCATTAC-3′ with an annealing temperature of 58 °C. The reactions were carried out on a CFX96 Touch™ Real-Time PCR Detection System (BioRad) and the results analysed using Bio-Rad CFX Manager. The expression levels of *wfs1a* and *wfs1b* were normalised to the housekeeping gene *ef1α*.

### PCR and gel electrophoresis

PCR reactions used MyTaq DNA Polymerase (Bioline) according to the manufacturer’s instructions. The primers used were the same as for the qPCR reactions, in addition to β-actinF 5′-CGAGCTGTCTTCCCATCCA-3′ and β-actin-R 5′-TCACCAACGTAGCTGTCTTTCTG-3′. The PCR products underwent electrophoresis in 1% (w/v) agarose (Bioline) gels made in 1× TAE buffer with Gel red (Merck). Bioline hyperladder IV was used and the gels were imaged using a GelDoc-IT Imaging system (UVP, Upland, USA).

### Acetylcholine esterase (AChE) activity assay

The motor neuron defects observed in mutant zebrafish led us to investigate whether there was also any difference in the total amount of neuronal tissue in mutant zebrafish compared with WT zebrafish. AChE is a cholinergic enzyme present in the post-synaptic junctions that hydrolyses the neurotransmitter acetylcholine. As AChE is expressed in most neuronal tissues, measuring the total amount of AChE in zebrafish embryo lysates provides an indirect measurement of total neuronal tissue. A modified AChE activity assay was used^49^. In brief, pooled embryos were homogenized in 0.5 ml ice-cold sodium phosphate buffer (0.1 M, pH 7.4, and 0.1% v/v Triton X-100). Homogenates were centrifuged for 15 min at 4 °C at 10,000×*g*. After quantification with a BCA assay, 0.3 mM DNTB and 0.45 mM AChE were added to 10 μg of protein and spectrophotometric readings at 405 nm was performed for 10 min.

### Fertility experiments

Zebrafish were group mated with 3 WT male and 3 female *wfs1*b^*−/−*^ knockouts per large breeding tank and data points were collected from 3 independent matings. Fifty randomly selected embryos were placed in E3 medium and then incubated overnight. The count of dead embryos was determined the following morning.

## Supplementary Information

Supplementary Information 1.

Supplementary Information 2.

Supplementary Information 3.

Supplementary Video 1.

Supplementary Video 1.

Supplementary Video 1.

Supplementary Video 2.

Supplementary Video 2.

Supplementary Video 2.

Supplementary Video 3.

Supplementary Video 3.

Supplementary Video 3.

## Abbreviations

AChE: Acetylcholine esterase
dpf: Days post-fertilisation
EZRC: European Zebrafish Resource Centre
F-Actin: Filamentous actin
hpf: Hours post-fertilisation
OA: Optic atrophy
OCT: Optical coherence tomography
OKR: Optokinetic response
qRT-PCR: Quantitative RT-PCR
RGC: Retinal ganglion cells
rpm: Revolutions per minute
RT-PCR: Reverse transcriptase PCR
UPR: Unfolded protein response
VEP: Visual evoked potentials
WS: Wolfram syndrome
WT: Wild-type
