# Supplementary material for: A mutant wfs1 zebrafish model of Wolfram syndrome manifesting visual dysfunction and developmental delay
Source: Sci Rep. 2021 Oct 14;11:20491. doi: 10.1038/s41598-021-99781-0 (PMC8516871; doi:10.1038/s41598-021-99781-0)
Supplement: Supplementary file 1 — Supplementary Information 1. [file 41598_2021_99781_MOESM1_ESM.docx]

## Supplementary Information

## A mutant *wfs1* zebrafish model of Wolfram syndrome manifesting visual dysfunction and developmental delay

### Cairns G^1,2^, Burté F^1^, Price R^1^, O’Connor E^3^, Toms M^4^, Mishra R^5^, Moosajee M^4,6,7^, Pyle A^8^, Sayer JA^1,9,10*^, Yu-Wai-Man P^4,5,6,11*^

Affiliations:

1. Institute of Genetic Medicine, Newcastle University, International Centre for Life, Newcastle upon Tyne, United Kingdom
2. Interdisciplinary School of Health Science, Faculty of Health Sciences, University of Ottawa.
3. Children's Hospital of Eastern Ontario Research Institute, University of Ottawa, Ottawa, Canada
4. UCL Institute of Ophthalmology, University College London, London, United Kingdom
5. John van Geest Centre for Brain Repair and MRC Mitochondrial Biology Unit, Department of Clinical Neurosciences, University of Cambridge, Cambridge, United Kingdom
6. Moorfields Eye Hospital NHS Foundation Trust, London, United Kingdom
7. Great Ormond Street Hospital for Children NHS Foundation Trust, London, United Kingdom
8. The Wellcome Centre for Mitochondrial Research, Translational and Clinical Research Institute, Newcastle University, Newcastle upon Tyne, United Kingdom
9. Department of Renal Medicine, Freeman Hospital, The Newcastle upon Tyne Hospitals NHS Foundation Trust, Newcastle upon Tyne, United Kingdom
10. National Institute for Health Research Newcastle Biomedical Research Centre, Newcastle upon Tyne, United Kingdom
11. Cambridge Eye Unit, Addenbrooke’s Hospital, Cambridge University Hospitals, Cambridge, UK

*Joint senior authors

### Corresponding Author:

Patrick Yu-Wai-Man

John van Geest Centre for Brain Repair and MRC Mitochondrial Biology Unit, Department of Clinical Neurosciences, University of Cambridge, Cambridge, United Kingdom; E-mail: [py237@cam.ac.uk](mailto:py237@cam.ac.uk).

## Supplementary Figures


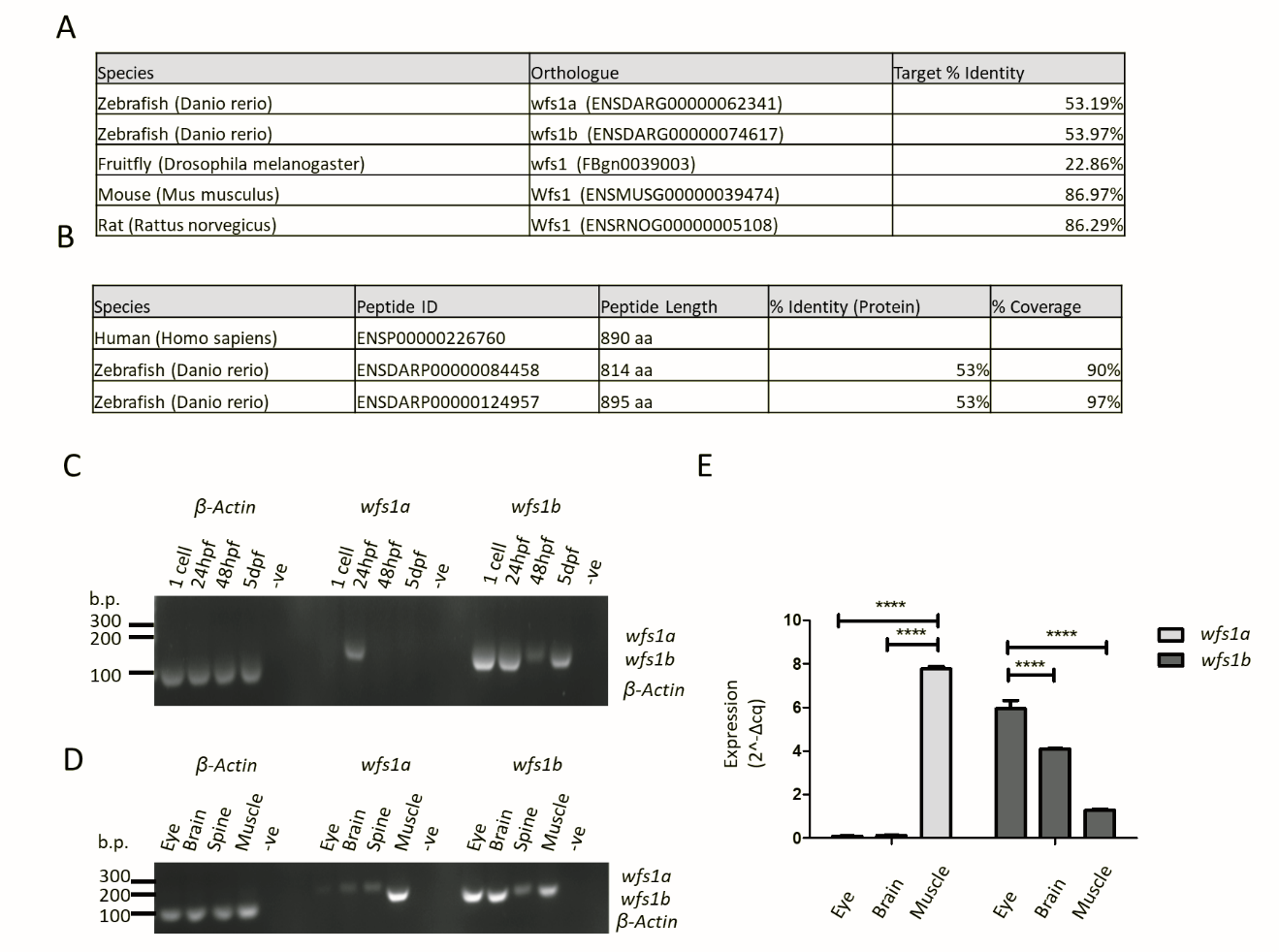


**Figure S1.** Expression of *wfs1* in embryonic and adult zebrafish. **A**: Sequence comparison between zebrafish and other species with the human *WFS1* gene. The table was created from Ensembl data. “Target % Identity” represents the degree of similarity to the human orthologue. **B**: Protein comparison between zebrafish and the human wolframin protein. **C**: Embryonic tissue expression of *wfs1a* and *wfs1b* in wild-type (WT) zebrafish up to 5 days post-fertilisation (dpf). The expected sizes are 100 bp for β-Actin, 175 bp for wfs1b and 181 bp for wfs1a. **D**: Expression of *wfs1a* and *wfs1b* in tissue lysates from 4-month-old zebrafish. **E**: qPCR quantification of adult tissue expression of *wfs1a* and *wfs1b* in tissue lysates from 4-month-old zebrafish. The expression levels were normalised to the housekeeping gene *ef1α*. cDNA expression was analysed on Bio-Rad CFX Manager and performed in triplicate. The plot showing the mean and standard error of the mean (SEM) was generated using Graphpad Prism (version 5) and the statistical significance was determined by the unpaired t-test ****p<0.0001. hpf: hours post-fertilisation; -ve: negative control.


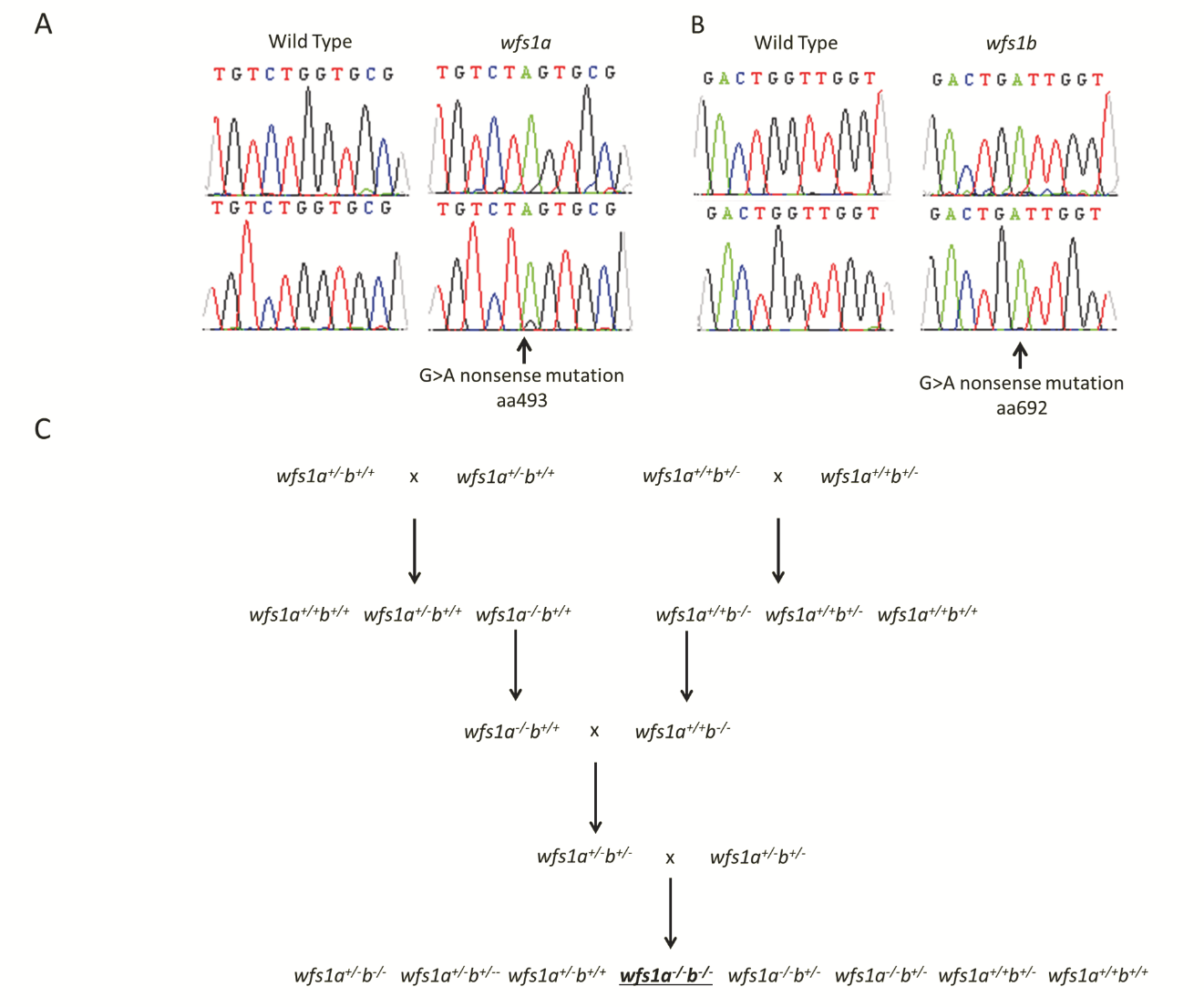


**Figure S2.** **A**: Sanger sequence chromatograms of the wild-type (WT) and *wfs1a* nonsense mutation. **B**: Sanger sequence chromatograms of the WT and *wfs1b* nonsense mutation. **C**: Diagram describing the breeding procedure used to generate single homozygous *wfs1a^-/-^* and *wfs1b^-/-^* lines, and a double knockout *wfs1a^-/-^b^-/-^* zebrafish.

***wfs1a***

1681 GACGTACTGTTTCCTGGTGCCGTACTTGGTCTGCTTTGTCTGGTGCGAGTTCTCTGTGGT 1740

1681 GACGTACTGTTTCCTGGTGCCGTACTTGGTCTGCTTTGTCTAGTGCGAGTTCTCTGTGGT 1740

431 --T--Y--C--F--L--V--P--Y--L--V--C--F--V--W--C--E--F--S--V--V 451

431 --T--Y--C--F--L--V--P--Y--L--V--C--F--V--Stop

***wfs1b***

1621 GGTTCAGCATGCGCCTTGGTGACTGGTTGGTAATGCATGTAGGTGTGCCTTGTGTTCTCT 1680

1621 GGTTCAGCATGCGCCTTGGTGACTGATTGGTAATGCATGTAGGTGTGCCTTGTGTTCTCT 1680

485 W--F--S--M--R--L--G--D--W--L--V--M--H--V--G--V--P--C--V--L-- 504

485 W--F--S--M--R--L--G--D--Stop

**Figure S3.** *wfs1* mutations and predicted amino acid sequence. The sequences were acquired from Ensembl genome browser 95 (<https://www.ensembl.org/>). The mutated nucleotide base is indicated in red.





**Figure S4.** The segmented line tool in ImageJ was used to measure axon length as demonstrated by the white line.





**Figure S5.** Quantification of eye area normalised to zebrafish length at 80 hpf. There were no significant differences between WT (mean = 0.020, n = 18), *wfs1*a*^-/-^* (mean = 0.020, n = 25) and *wfs1*b*^-/-^* (mean = 0.020, n = 25) embryos. Statistical significance was determined by One-Way ANOVA with Bonferroni multiple comparisons.





**Figure S6.** Quantitative PCR analysis of BiP expression in tissue lysates at 48 hpf. The mean values ± SEM have been normalised relative to WT embryos: WT (1.00 ± 0.80, n = 3), *wfs1*a*^-/-^* (0.63 ± 0.54, n = 3) and *wfs1*b*^-/-^* (0.34 ± 0.27, n = 3). cDNA expression was analysed on Bio-Rad CFX Manager and performed in triplicate. Statistical significance was determined by One-Way ANOVA with Bonferroni multiple comparisons.





**Figure S7.** Percentage of dead selected fertilised embryos at 24 hpf (n = 10). A total of 50 selected fertilised embryos were collected at 8 hpf. No significant difference was observed between fertilised controls (mean = 5.6%), *wfs1*a*^-/-^* knockouts (mean = 6.4%), and *wfs1*b*^-/-^* knockouts (mean = 11.1%). Data plots represent mean ± SEM. Statistical significance was determined by One-Way ANOVA with Bonferroni multiple comparisons.

## Supplementary Videos

**Supplementary Video 1.** Zebrafish spontaneous movement. Zebrafish from each condition were recorded for one minute per dish using a Leica stereomicroscope with a Chameleon digital camera (CMLN-13s2M) and spontaneous movements (coils within the chorion) were quantified. Fish strains used in this study include AB (wild-type), sa10021 (*wfs1a*) and sa16422 (*wfs1b*).

**Supplementary Video 2.** Zebrafish touch response. Single zebrafish were placed in fresh E3 medium on an illuminated stage and the back of the head was touched using a fine pipette tip to see the response to tactile stimulation. The videos were recorded using a Canon Legria hfr76 camera at 25 frames per second. Fish strains used in this study include AB (wild-type), sa10021 (*wfs1a*) and sa16422 (*wfs1b*).

**Supplementary Video 3.** Optokinetic response (OKR). Zebrafish were placed inside a variable speed rotating drum with a black and white grating. The grating was rotated at 8rpm for 30 seconds clockwise and 30 seconds anticlockwise. This protocol was repeated at 16 rpm. The videos were recorded using a Nikon camera attached to a stereomicroscope and eye movements were quantified. The videos are a cropped portion of the 16 rpm experiments on 12-month old zebrafish. Fish strains used in this study include AB (wild-type), sa10021 (*wfs1a*) and sa16422 (*wfs1b*).

## Original Blots


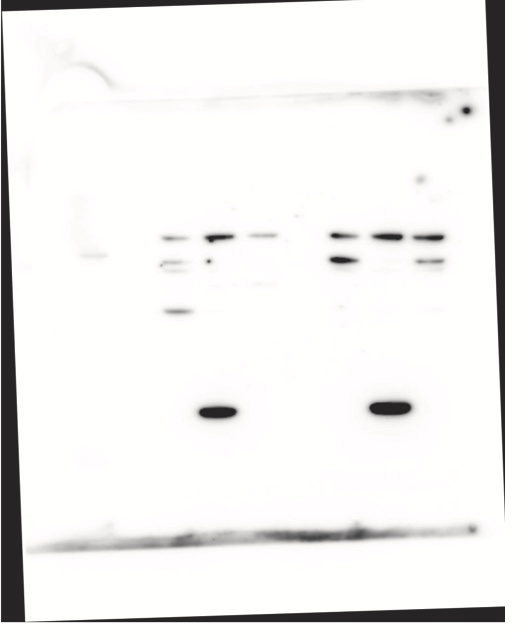


**Figure 2 – Panel C.** Western blot


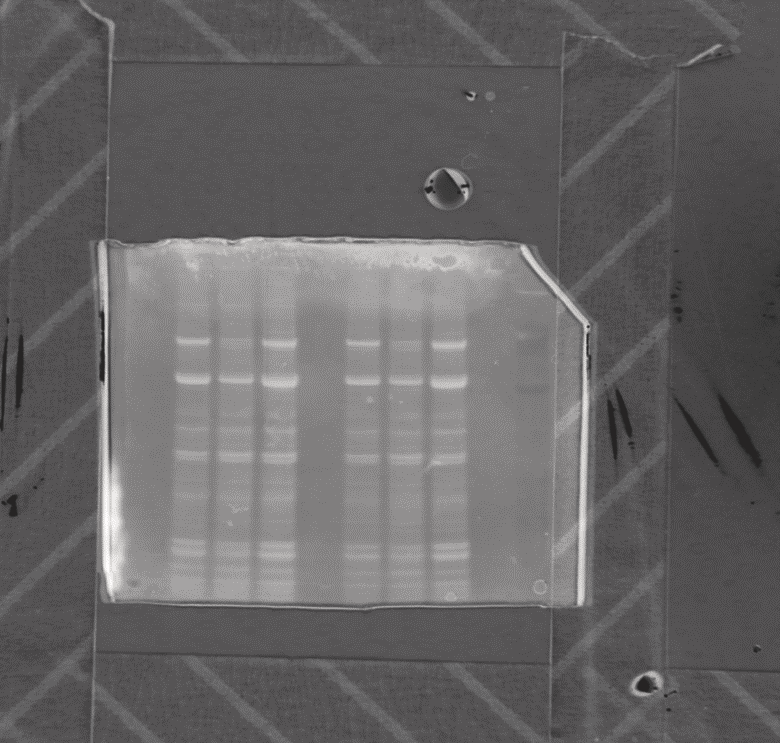


**Figure 2 – Panel C.** Coomassie staining


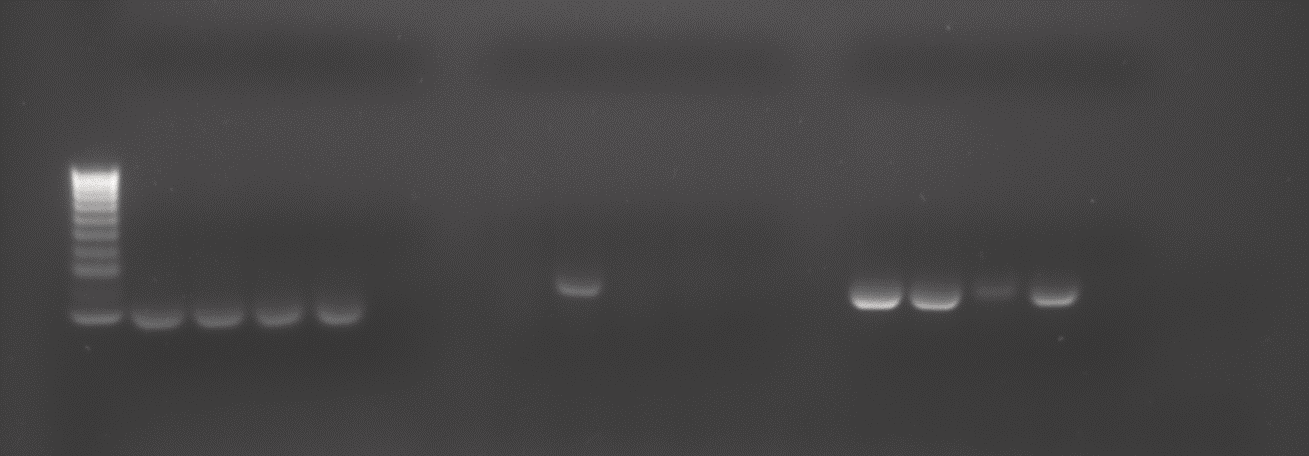


**Figure S1 – Panel C.** Full gel of quantitative RT-PCR products


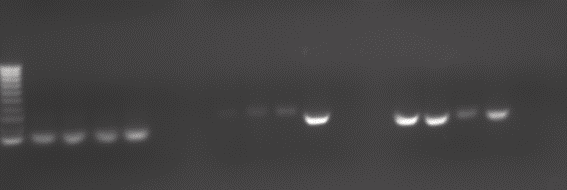


**Figure S1 – Panel D.** Full gel of quantitative RT-PCR products
